# Chronic Obstructive Pulmonary Disease With Asthma-Like Features in the General Population in China

**DOI:** 10.3389/fmed.2022.876240

**Published:** 2022-05-06

**Authors:** Kewu Huang, Kian Fan Chung, Ting Yang, Jianying Xu, Lan Yang, Jianping Zhao, Xiangyan Zhang, Chunxue Bai, Jian Kang, Pixin Ran, Huahao Shen, Fuqiang Wen, Yahong Chen, Tieying Sun, Guangliang Shan, Yingxiang Lin, Guodong Xu, Sinan Wu, Ying Wang, Xiaoying Gu, Ruiying Wang, Zhihong Shi, Yongjian Xu, Xianwei Ye, Yuanlin Song, Qiuyue Wang, Yumin Zhou, Wen Li, Liren Ding, Chun Wan, Wanzhen Yao, Yanfei Guo, Fei Xiao, Yong Lu, Xiaoxia Peng, Dan Xiao, Xiaoning Bu, Hong Zhang, Xiaolei Zhang, Li An, Shu Zhang, Zhixin Cao, Qingyuan Zhan, Yuanhua Yang, Lirong Liang, Wenjun Wang, Huaping Dai, Bin Cao, Jiang He, Chen Wang

**Affiliations:** ^1^Beijing Key Laboratory of Respiratory and Pulmonary Circulation Disorders, Department of Pulmonary and Critical Care Medicine, Beijing, China; ^2^Beijing Institute of Respiratory Medicine, Beijing, China; ^3^National Heart & Lung Institute, Imperial College London & Royal Brompton & Harefield NHS Trust, London, United Kingdom; ^4^National Center for Respiratory Medicine, Beijing, China; ^5^National Clinical Research Center for Respiratory Diseases, Beijing, China; ^6^Department of Pulmonary and Critical Care Medicine, Center of Respiratory Medicine, China-Japan Friendship Hospital, Beijing, China; ^7^Institute of Respiratory Medicine, Chinese Academy of Medical Sciences, Beijing, China; ^8^Department of Pulmonary and Critical Care Medicine, Shanxi Bethune Hospital, Taiyuan, China; ^9^Department of Pulmonary and Critical Care Medicine, The First Affiliated Hospital of Xi'an Jiaotong University, Xi'an, China; ^10^Department of Pulmonary and Critical Care Medicine, Tongji Hospital, Tongji Medical College, Huazhong University of Science and Technology, Wuhan, China; ^11^Department of Pulmonary and Critical Care Medicine, Guizhou Provincial People's Hospital, Guiyang, China; ^12^Department of Pulmonary and Critical Care Medicine, Zhongshan Hospital, Fudan University, Shanghai, China; ^13^Department of Pulmonary and Critical Care Medicine, The First Hospital of China Medical University, Shenyang, China; ^14^State Key Laboratory of Respiratory Disease, National Clinical Research Center for Respiratory Diseases, Guangzhou Institute of Respiratory Diseases, The First Affiliated Hospital, Guangzhou Medical University, Guangzhou, China; ^15^Department of Respiratory and Critical Care Medicine, The Second Affiliated Hospital of Zhejiang University, School of Medicine, Hangzhou, China; ^16^State Key Laboratory of Biotherapy of China and Department of Respiratory and Critical Care Medicine, West China Hospital of Sichuan University, Chengdu, China; ^17^Department of Respiratory and Critical Care Medicine, Peking University Third Hospital, Beijing, China; ^18^Department of Respiratory and Critical Care Medicine, Beijing Hospital, Beijing, China; ^19^National Center of Gerontology, Beijing Hospital, Beijing, China; ^20^Institute of Basic Medical Sciences, Chinese Academy of Medical Sciences; School of Basic Medicine, Peking Union Medical College, Beijing, China; ^21^Department of Clinical Research and Data Management, Center of Respiratory Medicine, China-Japan Friendship Hospital, Beijing, China; ^22^Center for Clinical Epidemiology and Evidence-Based Medicine, Beijing Children's Hospital, Capital Medical University, National Center for Children's Health, Beijing, China; ^23^Tobacco Medicine and Tobacco Cessation Center, Center of Respiratory Medicine, China-Japan Friendship Hospital, Beijing, China; ^24^WHO Collaborating Center for Tobacco Cessation and Respiratory Diseases Prevention, Beijing, China; ^25^Department of Epidemiology, Beijing Chao-Yang Hospital, Capital Medical University, Beijing, China; ^26^Department of Epidemiology, Tulane University School of Public Health and Tropical Medicine, New Orleans, LA, United States; ^27^Department of Respiratory Medicine, Capital Medical University, Beijing, China; ^28^Chinese Academy of Medical Sciences and Peking Union Medical College, Beijing, China

**Keywords:** asthma, bronchodilator response, chronic obstructive pulmonary disease, general population, wheeze

## Abstract

**Background:**

Patients with features of both asthma and chronic obstructive pulmonary disease (COPD) are seen commonly in the clinic but less is known in the general population. We investigated the prevalence and the heterogeneity of COPD with concomitant features of asthma in Chinese adult population.

**Methods:**

COPD was defined as post-bronchodilator ratio of forced expiratory volume in 1s (FEV_1_) to forced vital capacity of less than the lower limits of normal. COPD with concomitant features of asthma was defined as either COPD with asthma diagnosed by self-reported physician-diagnosis or by presence of current wheeze, or as COPD with high bronchodilator response (HBR) defined as an increase in FEV_1_ >15% and >400 ml after bronchodilator.

**Results:**

COPD with concomitant features of asthma was found in 1.62% (95% CI 1.31–2.00) of adults (≥20 years) or in 15.2% (95% CI 13.0–17.7) of COPD patients. Compared with COPD with HBR, COPD with asthma diagnosis or wheeze were older (61.8 ± 1.1 years vs. 47.4 ± 2.8 years, *P* < 0.001), and with a lower post-bronchodilator FEV_1_%pred (68.2 ± 2.3 vs. 96.6 ± 3.4, *P* < 0.001). Age, smoking status, biomass use and allergic rhinitis were associated with increasing prevalence of COPD with asthma diagnosis or wheeze, and had greater impaired health status, more comorbidities and more acute exacerbations in the preceding 12 months.

**Conclusions:**

COPD with concomitant features of asthma is common in people with COPD and those with COPD with asthma diagnosis or wheeze experience worse clinical severity than COPD with HBR. These findings will help toward the definition of the asthma-COPD overlap condition.

## Introduction

Chronic obstructive pulmonary disease (COPD) is a heterogeneous disease that is characterized by persistent respiratory symptoms and airflow limitation (1). 384 million adults worldwide have prevalent COPD and 3.2 million deaths were estimated to be due to COPD in 2015 (2). In China, COPD is reported in ~100 million of the adult population (3), and was the third leading cause of disability-adjusted life-years (DALYs) in 2017 (4). Between 15 and 55% of patients with COPD have some features of asthma with the proportion increasing with age (5, 6), a group that has been named previously as the asthma-COPD overlap (ACO) (6). These ACO patients represent a subset of COPD patients that had a poorer quality of life, a more rapid decline in pulmonary function, and a higher risk of exacerbation, compared to those with COPD alone (5, 6). However, these data have generally been obtained from hospital-based typical COPD patients, who were usually smokers and characterized by more severe disease and by those older than 40 years. In these studies, asthma was diagnosed from a previous physician-diagnosis of asthma or the occurrence of current asthma symptoms, such as wheezing. On the other hand, bronchial reversibility to a β-adrenergic agonist bronchodilator has been used as a typical trait of asthma, but its use to support an asthma diagnosis in ACO has been limited (7).

Previous studies have indicated that COPD is becoming more prevalent among young adults or never smokers (3, 8). In addition, the prevalence of COPD with concomitant features of asthma and its heterogeneity in the general population has not been reported previously.

To address these knowledge gaps, we used data from the national cross-sectional China Pulmonary Health (CPH) study, where COPD was defined by spirometric measurements (1), while asthma-like features were defined either as a previous physician-diagnosed asthma or with the presence of wheezing in the preceding 12 months, or by the presence of high bronchodilator responsiveness (HBR). Because this was a population-based study, we estimated the prevalence and examined the heterogeneity of COPD with concomitant features of asthma across the whole adult age range of over 20 years old.

## Methods

### Study Design and Population

The CPH study enrolled a nationally-representative sample of 57,779 adults aged 20 years or older in China between June 2012 and May 2015 and its study design and sampling methodology have been previously described (3). A multi-stage stratified cluster sampling procedure was utilized, which took into consideration geographic region, degree of urbanization, economic development status, and the sex and age distribution derived from the 2010 China census data (9). Briefly, in Stage one, we selected ten provinces, autonomous regions and municipalities (only regions below 1,500 meters of altitude were included), which represented the socioeconomic statuses and lifestyles of six major geographical regions in China. We randomly selected a large city, a midsize city, an economically-developed county, and an underdeveloped county from each province or autonomous region in Stage two. We randomly selected two urban districts from every city and two rural townships from every county in Stage three. We further randomly selected two urban residential communities or rural village communities (about 1,000–2,000 households) from the urban districts or rural townships, respectively in Stage four. Finally, we randomly selected individuals aged 20 years or older from the selected communities stratified by sex and age distribution based on the 2010 China census data. We selected only one participant from every household, without replacement. Temporary residents (living in their current residence <1 year); those who were physically incapable of taking a spirometry test; those admitted to hospital for any cardiac condition in the past 3 months, or with treated tuberculosis; or women who were pregnant or breastfeeding were excluded.

The questionnaire used in the CPH study was a revised form of the international BOLD study (10) and incorporated parts of questionnaires from the European Community Respiratory Health Survey (ECRHS) (11), the Allergic Rhinitis and its Impact on Asthma questionnaire (12), and the 12-item Short Form Health Survey (SF-12) (13). Trained interviewers administered the questionnaire with the aim of obtaining information regarding demographic characteristics, medical history, parental history of respiratory disease, and risk factors. Blood cell count data were also collected.

The study protocol was approved by the ethics committees of the Capital Medical University (Beijing, China) and all other participating institutes. Written informed consent was obtained from all participants.

### Lung Function Measurements

Trained and certified technicians carried out pulmonary function tests before or after bronchodilator inhalation (salbutamol 400 μg) on all participants using a MasterScreen Pneumo PC spirometer (CareFusion, Yorba Linda, CA, USA) according to a standard protocol (14). We did daily calibration with a 3 L syringe. Participants were required to do up to eight forced expiratory maneuvers until FVC and FEV_1_ were reproducible within 150 mL. We administered a bronchodilator (salbutamol 400 μg) by inhalation through a 500 mL spacer and repeated spirometry 20 min later, using the same criteria. Test results were stored in the spirometer and downloaded them daily to a central computer system. All the spirometric data were reviewed centrally by an expert panel on the basis of the criteria of the American Thoracic Society and European Respiratory Society (14). Poor-quality data were excluded.

### Definitions of COPD, Asthma and HBR

We defined COPD as a post-bronchodilator forced expiratory volume in one second (FEV_1_) to forced vital capacity (FVC) ratio of less than lower limits of normal (LLN) (1), using Chinese reference values to define airflow limitation (15). We defined asthma based on self-reported asthma diagnosis by a physician or by wheeze symptom in the preceding 12 months, which has been widely used in large-scale epidemiological studies (2, 16, 17). HBR was defined as an increase in FEV_1_ of >15% and >400 ml from baseline value, measured 20 min after inhalation of salbutamol (400 μg) (18).

**Figure 1:**
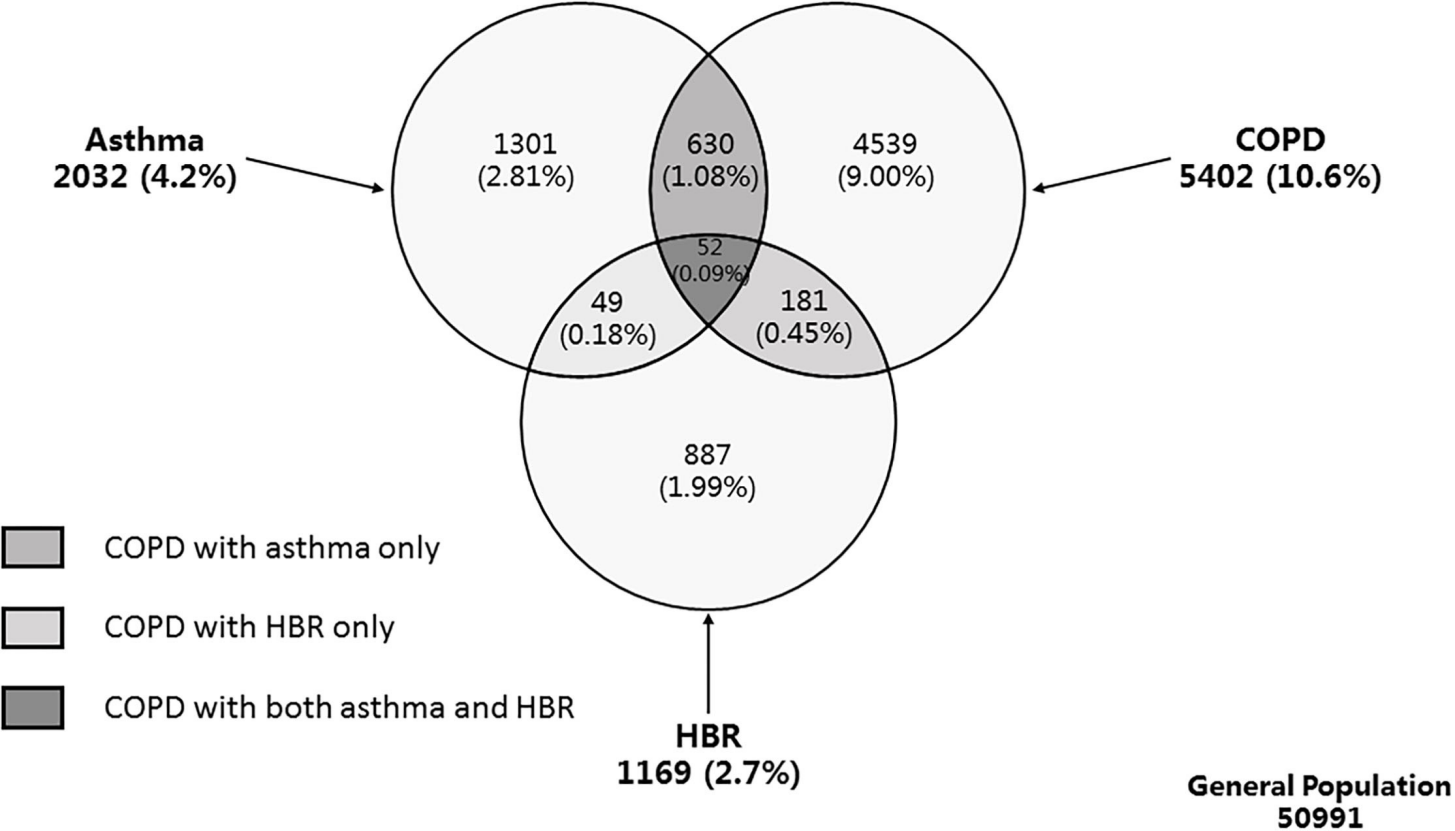
Venn diagram of weighted population prevalence of asthma, COPD, and HBR. Definition of abbreviations: COPD, chronic obstructive pulmonary disease; HBR, high bronchodilator responsiveness; LLN, lower limit of normal. Asthma was defined as physician-diagnosed asthma and/or wheeze in the preceding 12 months. COPD was defined as post-bronchodilator FEV_1_/FVC of less than LLN. HBR was defined as an increase in FEV_1_ of more than 15% and more than 400 ml from baseline, 20 min after bronchodilator with 400 μg salbutamol. COPD with concomitant features of asthma was defined as COPD with asthma or with HBR.

COPD with concomitant features of asthma was defined as COPD with asthma or with HBR. Therefore, we defined 3 types of COPD with concomitant features of asthma (Figure 1): (i) COPD with asthma only (ii) COPD with HBR only and (iii) COPD with both asthma and HBR. Finally, those COPD subjects without concomitant features of asthma were defined as ‘pure COPD’.

The definitions of ever-smoker, biomass use, exposure to ambient particulate matter with a diameter <2.5 μm (PM2.5), occupational exposure and allergic rhinitis have been previously reported (3).

### Statistical Analysis

All calculations were weighted to represent the general adult population aged 20 years or older in China, according to the 2010 population census (9). A technique appropriate for the complex survey design, the Taylor series linearization method, was used to calculate standard errors (SEs) (19). We used all participants for whom the variables of interest were available, and we did not impute missing data.

Characteristics of participants were compared across different groups using ANOVA or *t*-test for continuous variables and by χ^2^ test for categorical variables. The trend of prevalence by co-variables was tested using Cochrane-Armitage test. We examined the association between risk factors and different subtypes of COPD with concomitant features of asthma by multivariable logistic regression analyses.

All statistical analyses were performed with SUDAAN (Version 11.0; Research Triangle Institute, Research Triangle Park, NC) and SAS 9.4 (SAS Institute Inc., Cary, NC).

## Results

### Prevalence of COPD With Concomitant Features of Asthma

The final analysis included 50,991 subjects (21,446 men and 29,545 women) who completed the questionnaire survey and provided reliable pulmonary function tests before and after a bronchodilator. Of the 5,402 COPD patients, 863 with concomitant features of asthma were identified with a prevalence of 1.62% (95% CI 1.31–2.00). The prevalence for COPD with asthma only, COPD with HBR only, COPD with both asthma and HBR were 1.08% (95% CI 0.83–1.40), 0.45% (95% CI 0.30–0.68), and 0.09% (95% CI 0.05–0.15), respectively (Figure 1). The group of COPD with concomitant features of asthma consisted of 630 (66.7 %) with COPD with asthma only, 181 (27.8%) as COPD with HBR only, and 52 (5.5%) as COPD with both asthma and HBR. Compared with pure COPD, overall COPD people with concomitant features of asthma were older (57.1 years vs. 51.7 years, *P* < 0.001), showed a higher blood eosinophil percentage (3.3 vs. 2.7%, *P* = 0.015), had reduced lung function and impaired health status as measured by the SF-12 questionnaire, were more frequently treated with inhaled corticosteroids, inhaled bronchodilators and aminophylline, and experienced more exacerbations of respiratory symptoms in the preceding 12 months (*P* < 0.05 for all) (Table 1).

**Table 1:** Risk factors and clinical characteristics of COPD with concomitant features of asthma and pure COPD.

	**COPD (*n* = 5,402)**	**Overall COPD with concomitant features of asthma (*n* = 863)**	**Pure COPD (*n* = 4,539)**	***P-*value**
Men, %	3,200 (65.8%)	505 (64.5%)	2,695 (66.0%)	0.793
Age, years	52.5 (1.5)	57.1 (1.4)	51.7 (1.6)	<0.001
Body mass index, kg/m^2^	23.5 (0.2)	23.3 (0.2)	23.6 (0.2)	0.236
Ever smoker^‡^, %	2,518 (48.8%)	431 (50.8%)	2,087 (48.5%)	0.686
Biomass use, %	1,810 (38.3%)	334 (41.5%)	1,476 (37.8%)	0.188
Annual mean PM2.5 exposure (μg/m^3^)	72.7(4.1)	72.2(4.0)	72.7(4.1)	0.750
Occupational exposure^§^, %	1,461 (31.2%)	290 (31.5%)	1,171 (31.2%)	0.953
Allergic rhinitis	567 (13.2%)	170 (17.7%)	397 (12.4%)	0.097
Eosinophil percentage in peripheral blood (%)	2.8 (0.1)	3.3 (0.3)	2.7 (0.1)	0.015
**Lung function**				
Post-BD FEV_1_/FVC, %	63.4 (0.6)	58.5 (1.1)	64.3 (0.6)	<0.001
Post-BD FEV_1_% pred	83.1 (1.5)	76.4 (2.7)	84.3 (1.5)	0.002
**Short form (SF)-12 scores**				
PCS scores	50.7 (0.5)	46.0 (0.6)	51.5 (0.5)	<0.001
MCS scores	53.9 (0.4)	51.7 (0.5)	54.3 (0.4)	<0.001
**Comorbidities**				
Hypertension	535 (9.6%)	124 (13.2%)	411 (8.8%)	0.238
Coronary heart disease	130 (2.7%)	55 (4.8%)	75 (2.2%)	0.061
Diabetes	151 (2.0%)	31 (3.8%)	120 (1.6%)	0.333
**Medication usage**				
Inhaled corticosteroid	100 (3.9%)	75 (10.6%)	25 (0.6%)	0.010
Inhaled bronchodilator	146 (5.9%)	122 (15.3%)	24 (1.2%)	0.004
Aminophylline	166 (19.0%)	141 (21.4%)	25 (7.1%)	0.004
Systemic corticosteroid	85 (10.3%)	71 (11.1%)	14 (6.5%)	0.175
**Exacerbation of respiratory symptoms in the preceding 12 months**				
Emergency, %	269 (4.3%)	181 (18.8%)	88 (1.6%)	<0.001
Hospital admission, %	145 (2.5%)	101 (10.7%)	44 (1.0%)	<0.001

*Values are weighted and shown as number (%) or mean (SE). FEV_1_, forced expiratory volume in 1second; FVC, forced vital capacity; MCS, mental component summary; PCS, physical component summary; PM2.5, ambient particulate matter with a diameter <2.5 μm*.

‡ *Ever smoker was defined as having smoked equal to or >100 cigarettes in his/her lifetime*.

§ *Occupational exposure was defined as exposed to gas, smoke, chemical vapors or fumes in work above 3 months in his/her lifetime*.

The prevalence of overall COPD with concomitant features of asthma increased with age from 0.57% (95% CI 0.42–0.79) in individuals aged 20 to 39 years to 4.64% (95% CI 3.50–6.13) in those aged 60 years or older. Its prevalence was higher in ever-smokers compared to never-smokers (2.61 vs. 1.27%, *P* = 0.003). Similarly, the prevalence of overall COPD with concomitant features of asthma increased in those who used biomass fuel (2.25 vs. 1.44%, *P* = 0.016), as well as in those with allergic rhinitis (2.95 vs. 1.62%, *P* = 0.001). The effect of age, smoking status, biomass use, occupational exposure and allergic rhinitis on the prevalence was also observed in those with COPD with asthma only, but not in those with COPD with HBR only, with the exception of smoking status (Table 2).

**Table 2:** Age-specific and age-standardized prevalence of COPD with concomitant features of asthma in the general adult population.

	**Overall COPD with concomitant features of asthma (*n* = 863)**	**COPD with asthma only (*n* = 630)**	**COPD with HBR only (*n* = 181)**	**COPD with both asthma and HBR (*n* = 52)**
**Age (years)**				
20–39	0.57 (0.42, 0.79)	0.14 (0.07, 0.32)	0.40 (0.26, 0.63)	0.03 (0.01, 0.10)
40–59	1.42 (1.00, 2.01)	0.82 (0.60, 1.14)	0.43 (0.24, 0.79)	0.16 (0.08, 0.33)
≥60	4.64 (3.50, 6.13)	3.95 (2.96, 5.25)	0.62 (0.30, 1.28)	0.07 (0.02, 0.23)
*P*-value for trend	<0.001	<0.001	0.304	0.371
**Sex**				
Men	2.28 (1.66, 3.13)	1.50 (1.13, 2.00)	0.65 (0.37, 1.14)	0.13 (0.07, 0.24)
Women	1.21 (0.88, 1.68)	0.89 (0.57, 1.40)	0.27 (0.17, 0.44)	0.05 (0.02, 0.12)
*P*-value for trend	0.026	0.059	0.054	0.097
**Body mass index, kg/m** ^**2**^				
<18.5	2.67 (1.73, 4.09)	2.33 (1.49, 3.62)	0.34 (0.09, 1.25)	0.01 (0.00, 0.07)
18.5–24.9	1.80 (1.47, 2.20)	1.23 (0.96, 1.59)	0.49 (0.32, 0.77)	0.07 (0.03, 0.16)
≥25	1.55 (1.07, 2.22)	0.98 (0.69, 1.38)	0.46 (0.22, 0.93)	0.11 (0.05, 0.25)
*P*-value for trend	0.051	0.007	0.671	0.021
**Cigarette smoking**				
Never smoker	1.27 (0.93, 1.73)	0.83 (0.54, 1.27)	0.36 (0.21, 0.62)	0.08 (0.04, 0.14)
Ever smoker^‡^	2.61 (2.00, 3.40)	1.88 (1.43, 2.48)	0.62 (0.42, 0.93)	0.11 (0.05, 0.24)
*P*-value for trend	0.003	0.005	0.032	0.407
**Biomass use**				
Yes	2.25 (1.68, 3.03)	1.79 (1.40, 2.28)	0.41 (0.21, 0.81)	0.06 (0.02, 0.14)
No	1.44 (1.18, 1.75)	0.86 (0.70, 1.05)	0.48 (0.30, 0.77)	0.10 (0.06, 0.18)
*P*-value for trend	0.016	0.001	0.501	0.224
**Annual mean PM2.5 exposure (μg/m** ^**3**^ **)**				
<50	1.42 (0.69,2.91)	1.29 (0.59,2.81)	0.12 (0.06,0.24)	0.01 (0.00,0.10)
50–75	1.81 (1.30,2.54)	1.08 (0.81,1.45)	0.61 (0.34,1.09)	0.12 (0.06,0.23)
≥75	1.74 (1.36,2.22)	1.40 (1.00,1.95)	0.29 (0.16,0.50)	0.05 (0.03,0.11)
*P*-value for trend	0.556	0.839	0.071	0.052
**Occupational exposure^§^**				
Yes	2.08 (1.69, 2.56)	1.70 (1.29, 2.25)	0.31 (0.19, 0.51)	0.06 (0.03, 0.16)
No	1.61 (1.21, 2.14)	1.01 (0.78, 1.29)	0.51 (0.29, 0.88)	0.10 (0.05, 0.19)
*P*-value for trend	0.170	0.007	0.270	0.417
**Allergic rhinitis**				
Yes	2.95 (2.44, 3.57)	2.14 (1.69, 2.71)	0.56 (0.28, 1.12)	0.26 (0.07, 0.87)
No	1.62 (1.24, 2.11)	1.09 (0.82, 1.45)	0.46 (0.28, 0.76)	0.06 (0.03, 0.13)
*P*-value for trend	0.001	0.002	0.668	0.225

*Values are % (95% CI). HBR, highly bronchodilator responsiveness. PM2.5, ambient particulate matter with a diameter <2.5 μm*.

‡ *Ever smoker was defined as having smoked equal to or >100 cigarettes in his/her lifetime*.

§ *Occupational exposure was defined as exposed to gas, smoke, chemical vapors or fumes in work above 3 months in his/her lifetime*.

The age-specific and age-standardized prevalence of COPD with concomitant features of asthma in the ever-smoker is showed in Supplementary Table 1. The prevalence of COPD with concomitant features of asthma in ever-smoker aged ≥40 was 4.12% (95% CI 3.06–5.52).

### Distribution of Asthma Features in COPD

The proportion of overall COPD with concomitant features of asthma in COPD was 15.2% (95% CI 13.0–17.7), and it increased with age, from 9.8% (95% CI 6.7–14.2) in COPD people aged 20 to 39 years to 20.4% (95% CI 16.6–24.8) in those aged 60 years or older. Similarly, the proportion increased with the increasing severity of COPD, from 12.4% (95% CI 9.4–16.2) in GOLD stageI to 27.5% (95% CI 16.1–42.8) in GOLD stage IV (Table 3). Among the COPD group, proportion of COPD with asthma only was 10.1% (95% CI 8.1–12.7), COPD with HBR only was 4.3% (95% CI 2.9–6.1) and COPD with both asthma and HBR was 0.8% (95% CI 0.5–1.4). The proportion of these three subtypes did not differ in sex distribution or smoking status. However, the proportion of COPD with asthma increased with age (*P* < 0.001) and with decreasing lung function (*P* = 0.002), whereas the proportion of COPD with HBR decreased with age (*P* = 0.001) and with severity of lung dysfunction (*P* < 0.001) in the entire COPD population (Table 3). The distribution of asthma features in ever-smokers with COPD is shown in Supplementary Table 2. And among ever-smokers with COPD aged ≥40 years, overall COPD with asthma was 16.8% (95% CI 12.9–21.6) with the proportion of COPD with asthma only was 12.0% (95% CI 8.5–16.7).

**Table 3:** Proportion of COPD with concomitant features of asthma in COPD by sex, age, body mass index, smoking, allergic rhinitis and lung function.

	**Overall COPD with concomitant features of asthma (*n* = 863)**	**COPD with asthma only (*n* = 630)**	**COPD with HBR only (*n* = 181)**	**COPD with both asthma and HBR (*n* = 52)**
**Sex**				
Men	12.2 (8.5, 17.1)	6.7 (5.0, 8.8)	4.6 (2.4, 8.7)	0.9 (0.4, 1.7)
Women	14.8 (11.5, 18.8)	9.1 (6.4, 12.7)	4.9 (3.4, 7.2)	0.8 (0.3, 2.2)
*P*-value	0.413	0.237	0.869	0.888
**Age (years)**				
20–39	9.8 (6.7, 14.2)	2.3 (0.9, 5.7)	7.2 (4.8,10.6)	0.4 (0.1, 1.5)
40–59	13.4 (10.9, 16.3)	7.8 (5.9, 10.2)	4.0 (2.4, 6.5)	1.6 (0.9, 3.0)
≥60	20.4 (16.6, 24.8)	17.4 (14.2,21.2)	2.7 (1.5, 4.8)	0.3 (0.1, 1.3)
*P*-value	<0.001	<0.001	0.001	0.937
**Body mass index, kg/m** ^**2**^				
<18.5	18.1 (13.1, 24.5)	11.6 (7.9, 16.6)	6.4 (3.0, 13.1)	0.1 (0.0, 0.7)
18.5–24.9	13.9 (10.8, 17.7)	7.5 (5.6, 9.9)	5.7 (3.6, 8.7)	0.8 (0.3, 2.1)
≥25	13.6 (9.7, 18.6)	7.1 (5.4, 9.2)	5.5 (2.6, 10.9)	1.0 (0.5, 2.2)
*P*-value	0.1893	0.0539	0.7306	0.0223
**Smoking status**				
Ever smokers^‡^	11.8 (9.4, 14.7)	7.8 (5.7, 10.6)	3.2 (2.1, 4.9)	0.7 (0.3, 1.6)
Never smokers	13.8 (10.8, 17.4)	7.0 (5.0, 9.7)	5.8 (3.8, 8.7)	1.0 (0.6, 1.7)
*P*-value	0.360	0.655	0.061	0.365
**Allergic rhinitis**				
Yes	19.9 (13.5, 28.4)	14.7 (9.4, 22.1)	3.5 (2.0, 6.2)	1.7 (0.4, 7.3)
No	12.5 (10.0, 15.6)	6.4 (5.0, 8.1)	5.4 (3.8, 7.8)	0.7 (0.3, 1.6)
*P*-value	0.053	0.014	0.121	0.439
**Gold stage**				
I (FEV1 ≥80% predicted)	12.4 (9.4, 16.2)	5.1 (3.6, 7.1)	6.9 (4.5, 10.6)	0.4 (0.1, 1.0)
II (50% ≤ FEV1 <80% predicted)	12.4 (9.8, 15.6)	8.4 (6.6, 10.6)	1.8 (0.9, 3.6)	2.3 (1.0, 4.9)
III (30% ≤ FEV1 <50% predicted)	20.1 (13.2, 29.5)	18.5 (11.8, 27.8)	0.9 (0.1, 5.6)	0.8 (0.2, 3.4)
IV (FEV1 <30% predicted)	27.5 (16.1, 42.8)	27.5 (16.1, 42.8)	0.0 (.,.)	0.0 (.,.)
*P*-value	0.018	0.002	<0.001	0.061
**Total**	15.2 (13.0, 17.7)	10.1 (8.1, 12.7)	4.3 (2.9, 6.1)	0.8 (0.5, 1.4)

*Values are % (95% CI). FEV_1_, forced expiratory volume in 1 sd; FVC, forced vital capacity; HBR, highly bronchodilator responsiveness*.

‡ *Ever smoker was defined as having smoked equal to or >100 cigarettes in his/her lifetime*.

### Risk Factors for COPD With Features of Asthma

**Figure 2:**
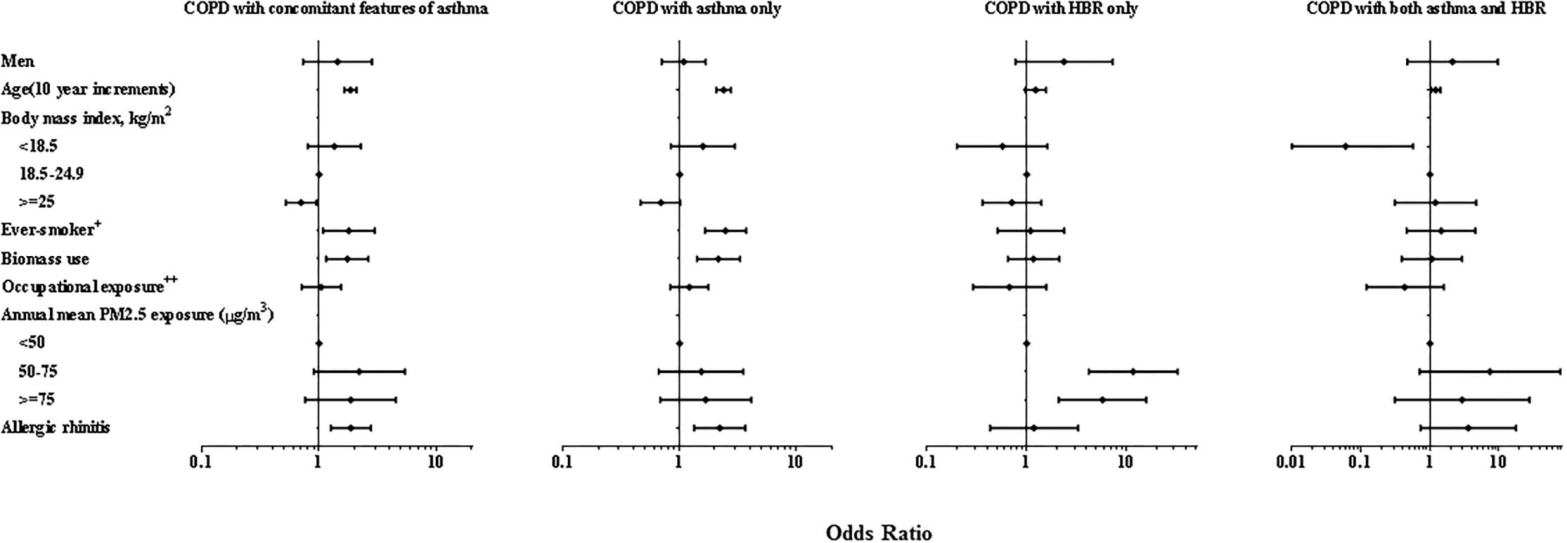
Forest plot of multiple adjusted odds ratios for COPD with concomitant features of asthma. Multivariable-adjusted analyses included all co-variable listed in the figure. ORs are represented by black blot while the 95% CI is presented by horizontal bars. 1.00 indicates reference values. HBR, highly bronchodilator responsiveness. PM2.5, ambient particulate matter with a diameter <2.5 μm. ^+^Ever smoker was defined as having smoked equal to or >100 cigarettes in his/her lifetime. ^++^Occupational exposure was defined as exposed to gas, smoke, chemical vapors or fumes in work above 3 months in his/her lifetime.

In the multivariate-adjusted analysis, the effects of age, smoking status, biomass use and allergic rhinitis on prevalence were also observed in overall COPD people with concomitant features of asthma and COPD with asthma only (*P* < 0.05 for all), but not in those with COPD with HBR only. However, PM2.5 exposure was a risk factor in those with COPD with HBR (*P* < 0.01) (Figure 2).

### Subtypes of COPD With Concomitant Features of Asthma

In contrast to COPD with HBR only, those with COPD and asthma only were older (61.8 ± 1.1 vs. 47.4 ± 2.8, *P* < 0.001), had a lower lung function (*P* < 0.001), and more impaired health state as measured by the PCS of the SF-12 questionnaire (43.3 vs. 52.1, *P* < 0.001) than COPD and HBR only. Compared with COPD and HBR only, COPD and asthma only had more comorbidities, including hypertension (15.6 vs. 4.1%, *P* = 0.024) and coronary heart disease (6.3 vs. 1.1%, *P* = 0.002). Among COPD and asthma only, 24.7% reported at least one emergency room visit and 13.4% reported at least one hospital admission in the preceding 12 months due to an exacerbation of respiratory symptoms, compared to 0.1% and 0.02% among COPD and HBR only, respectively (*P* < 0.001 for all) (Table 4); similar results were also observed in the ever-smokers with COPD people with features of asthma (Supplementary Table 3). Interestingly, compared with the pure COPD, COPD with HBR only had fewer emergency visits and hospital admissions in the preceding 12 months in the entire population (Supplementary Table 4) or in the ever-smokers (Supplementary Table 5).

**Table 4:** Comparison of clinical characteristics among the three subtypes of COPD with concomitant features of asthma in the general adult population.

	**COPD with asthma only (*n* = 630)**	**COPD with HBR only (*n* = 181)**	**COPD with both asthma and HBR (*n* = 52)**	***P-*** **value**		
				**COPD with asthma only vs. COPD with HBR only**	**COPD with asthma only vs. COPD with both asthma and HBR**	**COPD with HBR only vs. COPD with both asthma and HBR**
Men, %	341 (62.2%)	125 (68.6%)	39 (71.4%)	0.390	0.578	0.866
Age, years	61.8 (1.1)	47.4 (2.8)	48.0 (2.2)	<0.001	<0.001	0.883
Ever smoker^‡^	312 (54.4%)	90 (43.5%)	29 (43.0%)	0.165	0.438	0.968
Eosinophil percentage in peripheral blood (%)	3.3 (0.4)	3.0 (0.2)	3.8 (1.0)	0.554	0.625	0.453
**Lung function**						
Post-BD FEV_1_/FVC, %	55.2 (1.3)	66.1 (1.0)	59.7 (2.2)	<0.001	0.059	0.038
Post-BD FEV_1_% pred	68.2 (2.3)	96.6 (3.4)	73.8 (2.8)	<0.001	0.033	<0.001
**Short form (SF)-12 scores**						
PCS scores	43.3 (0.6)	52.1 (0.5)	47.5 (2.1)	<0.001	0.046	0.035
MCS scores	50.9 (0.8)	53.8 (0.9)	50.2 (2.5)	0.073	0.743	0.240
**Comorbidities**						
Allergic rhinitis	135 (18.7%)	20 (13.3%)	15 (29.5%)	0.361	0.532	0.378
Hypertension	103 (15.6%)	13 (4.1%)	8 (19.1%)	0.024	0.822	0.374
Coronary heart disease	48 (6.3%)	5 (1.1%)	2 (1.0%)	0.002	0.010	0.939
Diabetes	25 (4.6%)	4 (1.8%)	2 (1.3%)	0.350	0.255	0.729
**Medication use**						
Inhaled corticosteroid	63 (11.6%)	3 (1.6%)	9 (11.5%)	0.016	0.984	0.226
Inhaled bronchodilator	102 (15.7%)	3 (8.7%)	17 (21.2%)	0.105	0.693	0.406
Aminophylline	125 (20.7%)	2 (4.7%)	14 (31.8%)	0.008	0.288	0.016
Systemic corticosteroid	62 (11.9%)	2 (4.7%)	7 (2.2%)	0.118	<0.001	0.592
**Exacerbation of respiratory symptoms in the last 12 months**						
Emergency, %	162 (24.7%)	3 (0.1%)	16 (43.1%)	<0.001	0.145	0.001
Hospital admission, %	89 (13.4%)	1 (0.02%)	11 (32.4%)	<0.001	0.113	0.008

*Values are weighted and shown as number (%) or mean (SE). FEV_1_, forced expiratory volume in 1 s; FVC, forced vital capacity; HBR, highly bronchodilator responsiveness; MCS, mental component summary; PCS, physical component summary*.

‡ *Ever smoker was defined as having smoked equal to or >100 cigarettes in his/her lifetime*.

## Discussion

In this large survey of a nationally-representative sample of Chinese adults, we report that 1.62% of the Chinese adult population had COPD with concomitant features of asthma, representing 15.2% of people with COPD. 66.7% of those consisted of COPD with asthma only, while only 27.8% had COPD with HBR. Thus, COPD with asthma had a greater clinical severity than COPD with HBR in terms of greater degree of airflow obstruction, more impairment of quality of life, more exacerbations needing emergency room or hospitalization, and more comorbidities such as hypertension and coronary artery disease.

The most important finding is the distribution of these two types of COPD people with asthma features. Overall, COPD people with concomitant features of asthma were more prevalent in the older age group as has been reported previously (20, 21). We found that it is COPD with asthma only that reflects this increase in prevalence, while, surprisingly, in the COPD with HBR only group, there was an inverse relationship with increasing age. The decrease of bronchodilator responsiveness with age has also been reported in COPD patients (7) and in healthy subjects (22).

A criticism of studying younger individuals (<40 years) is that anyone defined as COPD according to our criteria would likely represent obstructive asthma, rather than clinically-diagnosed COPD which is usually made in those above 40 years old with a smoking history or exposed to other risk factors. However, there still exist the potential for such a COPD phenotype in the less than 40 year old age group (23), because there are other causes of airflow obstruction apart from chronic asthma such as poor lung development and bronchopulmonary dysplasia (24). We have not been able to further investigate these possibilities in our study.

We have chosen not to use the term ACO to define our COPD subjects with features of asthma because of the several definitions of ACO and the current lack of consensus (25). In our adult population-based study, we did not limit our analysis to those above 40 years old with a smoking history. One of the challenges of comparing the prevalence of ACO with previous publications (Supplementary Table 6) is that these studies have used different criteria for ACO, reflecting the lack of consensus on the ACO definition. While ACO has been defined mostly as physician-diagnosed asthma or current wheeze in individuals with post-bronchodilator airflow limitation, other studies used bronchodilator responsiveness in the diagnosis or as a separate definition on its own in those with post-bronchodilator airflow limitation. Other additional criteria have been used, such as a history of smoking with at least 10 pack-years or exposure to indoor or outdoor air pollution, an asthma diagnosis made before the age of 40 years, and presence of peripheral blood eosinophilia (5, 20, 25). We have preferred to link these criteria to the two defined subgroups of COPD with asthma features. The reported prevalence of ACO has ranged from 1.6% to 4.5% of the different population studied (20, 21, 26) compared to our reported prevalence of 1.62% in general population, or 4.12% in those of ever-smoker aged ≥40 years based on the ACO definition (25).

A significant bronchodilator response considered as a feature of asthma, has been used to distinguish asthma from COPD (18, 27) or the diagnosis of ACO (25, 28), and this recommendation has been widely used (26, 29–31). How this reversibility is related to symptom burden and phenotypic characteristics in COPD is unclear. Findings from population studies showed bronchodilator reversibility was not associated with symptom burden, exacerbations or health status in COPD patients (32). A cohort study for subjects with asthma or COPD from a chest clinic in Copenhagen showed that, after controlling for baseline FEV_1_, those of bronchodilator and corticosteroid reversibility, were significantly associated with better survival (33). Our present study shows that lung function and exacerbation events in people with COPD with HBR were better and less frequent than in COPD with asthma (Table 4), even better than in pure COPD (Supplementary Table 4), similar to the results of the Spanish study of Toledo-Pons and colleagues, where patients with COPD with HBR had better quality of life and fewer exacerbations than smoking asthmatics with airflow limitation, and patients with pure COPD (non-ACO) (29). The more favorable clinical outcome might, at least partly be contributed by the milder pulmonary function impairment and the younger age of those of COPD with HBR.

Previous study has suggested that patients with COPD demonstrating reversibility represent a subset of patients with evidence of increased airway eosinophilic inflammation (34), and that such patients may respond better to corticosteroid therapy (35). However, in our present study from the general population, we did not observe any difference on blood eosinophil percentage between COPD with HBR only, and pure COPD (Supplementary Table 4). There are at least two possible explanations for this inconsistency. First, the COPD people in the present study comes from the general population, not from hospital-based cohorts, and those of COPD with HBR did not include those with the previous physician-diagnosed asthma or current wheezing symptom. Second, blood eosinophil count has been reported not to correlate with airway eosinophilic inflammation in COPD patients (36). Thus, the airway pathophysiological characteristics of COPD with HBR need to be further explored.

Our study showed that COPD with asthma had a greater clinical severity than COPD with HBR in terms of greater degree of airflow obstruction, more impairment of quality of life, and more exacerbations needing emergency room or hospitalization. In addition, COPD with asthma had more comorbidities such as hypertension and coronary artery disease, which is consistent with the previous report from Zhu and colleagues, who have identified evidence of shared genetics between COPD and cardiac traits in a set of large-scale genome-wide association studies (GWAS) (37). These findings would indicate that those with COPD with asthma, rather than COPD with HBR would fit in more with the traditional label of ACO.

The multivariate-adjusted analysis showed that those with COPD and asthma had similar risk factors as we previously reported for COPD or asthma patients from the same population, such as age, smoking status, biomass use and allergic rhinitis (3, 17). Although the prevalence of COPD with HBR only was higher in ever-smokers than in never-smokers, we did not observe that smoking status was associated with COPD and HBR in the multivariate analysis, which is consistent with the report of Müller and colleagues who reported that smoking status did not influence bronchodilator reversibility of patients with COPD (38). We also did not observe that allergic rhinitis was a significant risk factor for COPD with HBR, while it was for those COPD with asthma. This was supported by the findings of a GWAS study that showed a strong genetic correlation between asthma and allergic diseases (39). We observed that higher exposure to PM2.5 was significantly associated the COPD with HBR; this may be partly contributed by pollution-induced airflow obstruction (40). Thus, our multivariate-adjusted analysis indicate that COPD with HBR was linked to risk factors distinct from those of COPD with asthma labeled as traditional ACO, implying that there are different origins or pathological mechanisms in those with COPD and HBR.

There are some limitations of our study. First, similar to other large-scale population-based surveys, the diagnosis of asthma was based mainly on current wheezing symptom using a standardized questionnaire, which could potentially have led to the misclassification of COPD as asthma because both diseases may present with wheezing, especially in people aged 40 years or older or in cigarette smokers. On the other hand, the diagnosis of COPD was based solely on spirometric measurements regardless of the presence of respiratory symptoms and the exposure of risk factors. Therefore, the spirometry-defined COPD in our study may not be clinically-diagnosed, especially in those of 20–40 years age, and in all likelihood would have been missed by clinicians. Second, the bronchodilator responsiveness defined as improvement of >12% and >200 ml after bronchodilator has previously been shown to be unstable with significant changes in this response between visits, as reported previously (41). This is a potential concern in this cross-sectional analysis, even though there was a strong effect of HBR in distinguishing asthma from COPD. Third, by design, the present cross-sectional study was not powered enough to capture clinical outcomes among the different subtypes of COPD people with asthma-like characteristics. A follow-up cohort study is needed to address this question.

## Conclusion

In a representative population of China, COPD with concomitant features of asthma is common in people with COPD and those with a previous physician diagnosis of asthma or the presence of current wheeze were more consistent with the traditional label of ACO, but those with COPD and HBR were not. Our findings contribute further to the definition of ACO in that HBR may not be a valid representation of asthma in this definition. Further progress will be achieved by identifying the treatable traits of chronic airways diseases without arbitrary disease labels (25, 29).

## Data Availability Statement

The raw data supporting the conclusions of this article will be made available by the authors, without undue reservation.

## Ethics Statement

The studies involving human participants were reviewed and approved by Capital Medical University (Beijing, China). The patients/participants provided their written informed consent to participate in this study.

## Author Contributions

KH, KC, JH, and CW conceived and designed the study. GX, SW, XG, GS, and JH analyzed the data. TY, JX, LY, JZ, XianZ, CB, JK, PR, HS, FW, YC, TS, YLin, YW, RW, ZS, YX, XY, YS, QW, YZ, WL, LD, CW, WY, YG, FX, YLu, XP, DX, XB, HZ, XiaoZ, LA, SZ, ZC, QZ, YY, LL, WW, HD, and BC contributed data/materials. KH, KC, and CW prepared the manuscript. All other authors revised the manuscript and approved of the final draft.

## Funding

CW was supported by the National Key R&D Program grant 2018YFC1315102 and 2016YFC0901100 from Ministry of Science and Technology of China and the Special Research Foundation for Public welfare of Health grant 201002008 from Ministry of Health of China. KH was partly supported by National Key R&D Program of China grant 2016YFC0901102 from Ministry of Science and Technology of China, the National Natural Science Foundation of China grant 81870032 and the ‘Summit’ talent training program grant DFL20190301 from Beijing Hospital Authority.

## Conflict of Interest

The authors declare that the research was conducted in the absence of any commercial or financial relationships that could be construed as a potential conflict of interest.

## Publisher's Note

All claims expressed in this article are solely those of the authors and do not necessarily represent those of their affiliated organizations, or those of the publisher, the editors and the reviewers. Any product that may be evaluated in this article, or claim that may be made by its manufacturer, is not guaranteed or endorsed by the publisher.
