# Supplementary material for: Chronic Obstructive Pulmonary Disease With Asthma-Like Features in the General Population in China
Source: Front Med (Lausanne). 2022 May 6;9:876240. doi: 10.3389/fmed.2022.876240 (PMC9120624; doi:10.3389/fmed.2022.876240)
Supplement: Supplementary file 1 [file Data_Sheet_1.doc]

**Supplementary Appendix**

**Supplement to:**

**Chronic obstructive pulmonary disease with asthma-like features in the general population in China**

**TABLE E1 Age-specific and age-standardized prevalence of COPD with concomitant features of asthma in the ever-smoker**

|  | **Overall COPD with concomitant features of asthma**  **(n=431)** | **COPD with asthma only**  **(n=312)** | **COPD with HBR only**  **(n=90)** | **COPD with both asthma and HBR**  **(n=29)** |
| --- | --- | --- | --- | --- |
| **Age (years)** |  |  |  |  |
| 20-39 | 0.36 (0.16,0.80) | 0.17 (0.04,0.71) | 0.18 (0.07,0.50) | 0.00 (0.00,0.01) |
| 40-59 | 2.27 (1.55,3.31) | 1.46 (0.83,2.56) | 0.55 (0.36,0.83) | 0.26 (0.12,0.59) |
| ≥60 | 7.68 (5.57,10.5) | 6.02 (4.72,7.64) | 1.63 (0.75,3.51) | 0.03 (0.01,0.10) |
| *P* value for trend | <0.001 | <0.001 | 0.033 | 0.079 |
| **Sex** |  |  |  |  |
| Men | 2.76 (2.09,3.65) | 1.97 (1.48,2.61) | 0.68 (0.44,1.06) | 0.11 (0.05,0.24) |
| Women | 1.90 (1.13,3.19) | 1.86 (1.10,3.14) | 0.01 (0.00,0.13) | 0.03 (0.00,0.29) |
| *P* value for trend | 0.190 | 0.857 | <0.001 | 0.092 |
| **Body mass index, kg/m^2^** |  |  |  |  |
| < 18.5 | 3.84 (2.61,5.62) | 2.88 (2.12,3.90) | 0.96 (0.29,3.15) | 0.00 (., . ) |
| 18.5-24.9 | 2.56 (1.93,3.38) | 1.81 (1.35,2.41) | 0.58 (0.36,0.92) | 0.17 (0.08,0.38) |
| ≥25 | 2.65 (1.39,4.99) | 1.84 (1.09,3.11) | 0.76 (0.27,2.17) | 0.04 (0.01,0.16) |
| *P* value for trend | 0.285 | 0.112 | 0.771 | 0.163 |
| **Biomass use** |  |  |  |  |
| Yes | 3.41 (2.49,4.67) | 2.84 (1.99,4.04) | 0.52 (0.29,0.93) | 0.05 (0.01,0.18) |
| No | 2.01 (1.70,2.37) | 1.20 (0.95,1.52) | 0.67 (0.35,1.31) | 0.13 (0.06,0.30) |
| *P* value for trend | 0.007 | 0.007 | 0.588 | 0.181 |
| **Annual mean PM2.5 exposure (µg/m^3^)** |  |  |  |  |
| <50 | 2.35 (0.95,5.70) | 2.14 (0.82,5.50) | 0.18 (0.06,0.56) | 0.03 (0.00,0.30) |
| 50-75 | 2.86 (1.94,4.18) | 1.93 (1.30,2.85) | 0.76 (0.43,1.33) | 0.17 (0.07,0.42) |
| ≥75 | 2.45 (1.51,3.97) | 1.91 (1.15,3.15) | 0.51 (0.27,0.94) | 0.04 (0.01,0.14) |
| *P* value for trend | 0.927 | 0.833 | 0.080 | 0.831 |
| **Occupational exposure^§^** |  |  |  |  |
| Yes | 2.91 (2.23,3.80) | 2.50 (1.84,3.40) | 0.35 (0.23,0.55) | 0.06 (0.01,0.25) |
| No | 2.39 (1.69,3.37) | 1.52 (1.10,2.09) | 0.73 (0.43,1.24) | 0.14 (0.06,0.31) |
| *P* value for trend | 0.326 | 0.035 | 0.077 | 0.166 |
| **Allergic rhinitis** |  |  |  |  |
| Yes | 4.17 (2.84,6.09) | 2.59 (1.82,3.69) | 1.39 (0.49,3.89) | 0.19 (0.08,0.45) |
| No | 2.48 (1.85,3.33) | 1.82 (1.32,2.49) | 0.56 (0.35,0.91) | 0.11 (0.05,0.24) |
| *P* value for trend | 0.049 | 0.181 | 0.267 | 0.291 |

Values are % (95% CI). Abbreviations: HBR, highly bronchodilator responsiveness. PM2.5, ambient particulate matter with a diameter less than 2.5 µm.

^‡^Ever smoker was defined as having smoked equal to or greater than 100 cigarettes in his/her lifetime.

^§^Occupational exposure was defined as exposed to gas, smoke, chemical vapors or fumes in work above 3 months in his/her lifetime.

**TABLE E2 Proportion of COPD with concomitant features of asthma in the ever-smoker with COPD by sex, age, body mass index, allergic rhinitis, smoking and lung function**

|  | **Overall COPD with concomitant features of asthma**  **(n=431)** | **COPD with asthma only**  **(n=312)** | **COPD with HBR only**  **(n=90)** | **COPD with both asthma and HBR**  **(n=29)** |
| --- | --- | --- | --- | --- |
| **Sex** |  |  |  |  |
| Men | 11.8 (9.4, 14.9) | 7.8 (5.7, 10.6) | 3.3 (2.2, 5.1) | 0.7 (0.3, 1.6) |
| Women | 18.4 (11.9, 27.3) | 18.1 (11.8, 26.8) | 0.1 (0.0, 0.7) | 0.2 (0.0, 2.4) |
| *P* value | 0.125 | 0.014 | <0.001 | 0.156 |
| **Age (years)** |  |  |  |  |
| 20-39 | 4.2 (1.6, 10.6) | 1.5 (0.4, 6.1) | 2.6 (0.7, 9.1) | 0.0 (0.0, 0.1) |
| 40-59 | 14.1 (10.3, 19.0) | 9.2 (5.2, 15.7) | 3.2 (2.4, 4.1) | 1.7 (0.7, 4.0) |
| ≥60 | 22.1 (17.3, 27.8) | 17.5 (14.3, 21.2) | 4.5 (2.5, 8.1) | 0.1 (0.0, 0.3) |
| *P* value | <0.001 | <0.001 | 0.388 | 0.112 |
| **Body mass index, kg/m^2^** |  |  |  |  |
| < 18.5 | 21.7 (12.7, 34.5) | 9.1 (6.0, 13.6) | 12.6 (5.5, 26.3) | 0.0 (., . ) |
| 18.5-24.9 | 11.3 (8.0, 15.8) | 7.1 (4.9, 10.3) | 3.1 (1.5, 6.3) | 1.1 (0.4, 2.8) |
| ≥25 | 12.7 (8.6, 18.2) | 9.0 (5.9, 13.5) | 3.4 (2.0, 5.7) | 0.3 (0.1, 0.9) |
| *P* value | 0.1468 | 0.9639 | 0.0725 | 0.1301 |
| **Allergic rhinitis** |  |  |  |  |
| Yes | 18.0 (14.0, 22.9)) | 11.7 (7.7, 17.6) | 5.1 (2.5, 10.2) | 1.1 (0.4, 3.4) |
| No | 10.9 (8.4, 13.9 | 7.1 (5.1, 10.0) | 3.0 (1.8, 5.1) | 0.7 (0.3, 1.7) |
| *P* value | 0.007 | 0.097 | 0.350 | 0.505 |
| **GOLD stage** |  |  |  |  |
| Ⅰ (FEV1 ≥80% predicted) | 10.5 (7.5, 14.6) | 5.3 (3.2, 8.5) | 4.7 (2.8, 8.0) | 0.5 (0.1, 2.0) |
| Ⅱ (50% ≤ FEV1 < 80% predicted) | 11.0 (7.6, 15.5) | 8.2 (5.2, 12.7) | 1.8 (0.9, 3.6) | 1.0 (0.3, 2.7) |
| Ⅲ (30% ≤ FEV1 < 50% predicted) | 19.8 (11.1, 32.8) | 18.5 (10.3, 31.1) | 0.2 (0.0, 1.5) | 1.1 (0.2, 5.7) |
| Ⅳ (FEV1 <30% predicted) | 20.5 (11.4, 34.1) | 20.5 (11.4, 34.1) | 0.0 (., . ) | 0.0 (., . ) |
| *P* value | 0.082 | 0.016 | <0.001 | 0.373 |
| **Total** | 15.8 (12.6, 19.6) | 11.3 ( 8.7, 14.5) | 3.8 ( 2.7, 5.3) | 0.7 ( 0.3, 1.7) |

Values are % (95% CI). Abbreviations: FEV_1_, forced expiratory volume in one second; FVC, forced vital capacity

**TABLE E3 Comparison of clinical characteristics among the three subtypes of COPD with concomitant features of asthma in the ever-smokers**

|  | **COPD with asthma only (n=312)** | **COPD with HBR only (n=90)** | **COPD with both asthma and HBR (n=29)** | ***P* value** | | |
| --- | --- | --- | --- | --- | --- | --- |
|  |  |  |  | **COPD with asthma only *vs* COPD with HBR only** | **COPD with asthma only *vs* COPD with both asthma and HBR** | **COPD with HBR only *vs* COPD with both asthma and HBR** |
| Men, % | 276 (94.6%) | 88 (99.8%) | 28 (98.8%) | 0.057 | 0.144 | 0.383 |
| Age, years | 62.0 (1.4) | 59.5 (4.1) | 49.3 (2.1) | 0.599 | <0.001 | 0.034 |
| Eosinophil percentage in peripheral blood (%) | 3.1 (0.3) | 3.2 (0.7) | 4.3 (1.7) | 0.903 | 0.509 | 0.539 |
| **Lung function** |  |  |  |  |  |  |
| Post-BD FEV_1_/FVC, % | 54.0 (2.2) | 62.9 (1.3) | 60.5 (3.4) | 0.003 | 0.123 | 0.476 |
| Post-BD FEV_1_% pred | 67.4 (3.0) | 96.9 (5.9) | 75.6 (4.2) | <0.001 | 0.018 | 0.001 |
| **Short form (SF)-12 scores** |  |  |  |  |  |  |
| PCS scores | 43.4 (0.8) | 51.9 (0.9) | 48.4 (1.7) | <0.001 | 0.012 | 0.064 |
| MCS scores | 49.7 (1.0) | 55.4 (1.0) | 51.4 (3.1) | 0.002 | 0.607 | 0.235 |
| **Comorbidities** |  |  |  |  |  |  |
| Allergic rhinitis | 53 (12.7%) | 12 (20.4%) | 8 (15.9%) | 0.505 | 0.744 | 0.733 |
| Hypertension | 56 (19.2%) | 8 (7.5%) | 4 (10.7%) | 0.042 | 0.398 | 0.774 |
| Coronary heart disease | 30 (8.5%) | 3 (1.7%) | 0 (0.0%) | 0.004 | 0.001 | 0.352 |
| Diabetes | 15 (6.4%) | 2 (2.9%) | 1 (1.7%) | 0.361 | 0.186 | 0.689 |
| **Medication use** |  |  |  |  |  |  |
| Inhaled corticosteroid | 21 (12.0%) | 1 (1.8%) | 4 (2.2%) | 0.061 | 0.070 | 0.890 |
| Inhaled bronchodilator | 41 (14.3%) | 2 (7.1%) | 11 (21.7%) | 0.265 | 0.628 | 0.350 |
| Aminophylline | 60 (24.1%) | 0 (0.0%) | 7 (10.7%) | <0.001 | 0.148 | 0.260 |
| Systemic corticosteroid | 24 (11.6%) | 0 (0.0%) | 4 (1.8%) | 0.011 | 0.018 | 0.226 |
| **Exacerbation of respiratory symptoms in the last 12 months** |  |  |  |  |  |  |
| Emergency, % | 69 (25.1%) | 1 (0.2%) | 8 (24.9%) | <0.001 | 0.987 | 0.027 |
| Hospital admission, % | 38 (12.6%) | 0 (0.0%) | 7 (22.7%) | <0.001 | 0.290 | 0.023 |

Values are weighted and shown as number (%) or mean (SE). Abbreviations: FEV_1_, forced expiratory volume in one second; FVC, forced vital capacity; HBR, highly bronchodilator responsiveness; MCS, mental component summary; PCS, physical component summary.

**TABLE E4** Comparison of clinical features between COPD with HBR only and pure COPD in the general adult population

|  | **COPD with HBR only**  **(n=181)** | **Pure COPD**  **(n=4539)** | ***P* value** |
| --- | --- | --- | --- |
| Men, % | 125 (68.6%) | 2695 (66.0%) | 0.740 |
| Age, years | 47.4 (2.8) | 51.7 (1.6) | 0.095 |
| Ever smoker‡ | 90 (43.5%) | 2087 (48.5%) | 0.467 |
| Eosinophil percentage in peripheral blood (%) | 3.0 (0.2) | 2.7 (0.1) | 0.408 |
| **Lung function** |  |  |  |
| Post-BD FEV_1_/FVC, % | 66.1 (1.0) | 64.3 (0.6) | 0.058 |
| Post-BD FEV_1_% pred | 96.6 (3.4) | 84.3 (1.5) | <0.001 |
| **Short form (SF)-12 scores** |  |  |  |
| PCS scores | 52.1 (0.5) | 51.5 (0.5) | 0.283 |
| MCS scores | 53.8 (0.9) | 54.3 (0.4) | 0.552 |
| **Comorbidities** |  |  |  |
| Allergic rhinitis | 20 (13.3%) | 397 (12.4%) | 0.796 |
| Hypertension | 13 (4.1%) | 411 (8.8%) | 0.076 |
| Coronary heart disease | 5 (1.1%) | 75 (2.2%) | 0.373 |
| Diabetes | 4 (1.8%) | 120 (1.6%) | 0.863 |
| **Medication use** |  |  |  |
| Inhaled corticosteroid | 3 (1.6%) | 25 (0.6%) | 0.530 |
| Inhaled bronchodilator | 3 (8.7%) | 24 (1.2%) | 0.185 |
| Aminophylline | 2 (4.7%) | 25 (7.1%) | 0.686 |
| Systemic corticosteroid | 2 (4.7%) | 14 (6.5%) | 0.759 |
| **Exacerbation of respiratory symptoms in the last 12 months** |  |  |  |
| Emergency, % | 3 (0.1%) | 88 (1.6%) | 0.002 |
| Hospital admission, % | 1 (0.02%) | 44 (1.0%) | 0.035 |

Values are weighted and shown as number (%) or mean (SE). Abbreviations: FEV_1_, forced expiratory volume in one second; FVC, forced vital capacity; HBR, highly bronchodilator responsiveness; MCS, mental component summary; PCS, physical component summary.

^‡^Ever smoker was defined as having smoked equal to or greater than 100 cigarettes in his/her lifetime.

**TABLE E5 Comparison of clinical features between COPD with HBR only and pure COPD in the ever-smokers**

|  | **COPD with HBR only (n=90)** | **Pure COPD**  **(n=2087)** | ***P* value** |
| --- | --- | --- | --- |
| Men, % | 88 (99.8%) | 1961 (95.0%) | 0.084 |
| Age, years | 59.5 (4.1) | 53.4 (1.5) | 0.174 |
| Eosinophil percentage in peripheral blood (%) | 3.2 (0.7) | 2.7 (0.2) | 0.544 |
| **Lung function** |  |  |  |
| Post-BD FEV_1_/FVC, % | 62.9 (1.3) | 62.8 (0.9) | 0.958 |
| Post-BD FEV_1_% pred | 96.9 (5.9) | 83.6 (1.6) | 0.003 |
| **Short form (SF)-12 scores** |  |  |  |
| PCS scores | 51.9 (0.9) | 51.6 (0.3) | 0.684 |
| MCS scores | 55.4 (1.0) | 54.6 (0.3) | 0.428 |
| **Comorbidities** |  |  |  |
| Allergic rhinitis | 12 (20.4%) | 173 (9.7%) | 0.216 |
| Hypertension | 8 (7.5%) | 178 (8.5%) | 0.855 |
| Coronary heart disease | 3 (1.7%) | 23 (1.0%) | 0.684 |
| Diabetes | 2 (2.9%) | 50 (1.4%) | 0.604 |
| **Medication use** |  |  |  |
| Inhaled corticosteroid | 1 (1.8%) | 8 (0.4%) | 0.489 |
| Inhaled bronchodilator | 2 (7.1%) | 14 (1.7%) | 0.361 |
| Aminophylline | 0 (0.0%) | 12 (6.4%) | 0.067 |
| Systemic corticosteroid | 0 (0.0%) | 6 (3.0%) | 0.195 |
| **Exacerbation of respiratory symptoms in the last 12 months** |  |  |  |
| Emergency, % | 1 (0.2%) | 40 (1.6%) | 0.015 |
| Hospital admission, % | 0 (0.0%) | 17 (0.8%) | 0.022 |

Values are weighted and shown as number (%) or mean (SE). Abbreviations: FEV_1_, forced expiratory volume in one second; FVC, forced vital capacity; HBR, highly bronchodilator responsiveness; MCS, mental component summary; PCS, physical component summary.

**TABLE E6 ACO prevalence from different studies**

| **First author, publication year** | **Study Name** | **Type of Study** | **Country** | **Population** | **Sample Size** | **ACO Definition** | **ACO Subject** | **ACO Prevalence** | | |
| --- | --- | --- | --- | --- | --- | --- | --- | --- | --- | --- |
|  |  |  |  |  |  |  |  | **in general population** | | **in COPD** |
| De Marco R, 2013 (1) | GEIRD study | nested multi case-control | Italy | 20-44, 45-64, 65-84 years, general population | 20–44 years 5163,  45–64 years 2167,  65–84 years 1030 | Asthma: physician diagnosed, collected by questionnaire; COPD: physician diagnosed, collected by questionnaire; ACO was not defined. | NA | 20–44:1.6%  45–64:2.1%  65–84:4.5% | | 20–44:33%  45–64:27%  65–84:25% |
| Guerriero M, 2019 (2) | A study in Verona | cross-sectional | Northern Italy | 20-79 years, general population | 1,236 | ACO: 1. AO (FEV_1_/FVC < LLN), 2. highly positive bronchodilator test (≥15% increase in FEV_1_ and FVC ≥400 mL), and 3. personal self-reported history of physician diagnosed asthma and atopy. | 26 | 2.1% | | 18.8% |
| Morgan B W, 2019 (3) | CRONICAS study,  PRISA study,  the Bangladesh study,  LiNK study | cross-sectional | 6 low- and middle-income countries | 35-92 years, general population | 11, 923 | ACO: having both COPD and asthma;  COPD: post-BD FEV_1_/FVC ＜LLN;  asthma: as wheeze or medication use in 12 months or self-reported physician diagnosis | 450 | 3.8% | | 43.8% |
| Miravitlles M, 2013 (4) | EPI-SCAN study | cross-sectional | Spain | 40-80 years, general population | 3,885 | ACO: 1. post-BD FEV_1_/FVC ratio of < 0.70, 2. answered affirmatively that they had been previously diagnosed with asthma | 67 | 1.7% | | 17.4% |
| Kumbhare S, 2016 (5) | BRFSS study | cross-sectional | USA | ≥ 35 years, general population | 9,0851 | ACO: 1. physician diagnosed COPD, collected by questionnaire; 2. physician diagnosed asthma and currently diagnosed with asthma, collected by questionnaire | 2,594 | 3.2% | | 35.0% |
| Menezes A M B, 2014 (6) | PLATINO study | Longitude | Latin America | general population | 5,044 | ACO: the combination of COPD and asthma; COPD: post-BD FEV_1_/FVC ratio of ≤ 0.70; asthma: presence of wheezing in the last year and a minimum post-BD increase in FEV_1_ or FVC of 12% and 200 mL | 89 | 1.8% | | 13.1% |
| Krishnan J A, 2019 (7) | REG’s ACO Working Group conducted study | cross-sectional | UK | ≥40 years old and with ≥2 outpatient primary care visits over a 2-year period in the UK Optimum Patient Care Research Database | 2,165 | ACO: 1. age ≥ 40 years, 2. current or former smoking, 3. post-BD airflow limitation (FEV_1/_FVC <0.7), and 4. ≥12% and ≥200 mL reversibility in post-BD FEV_1_ | 444 | NA | | 31.5% |
| Barrecheguren M, 2015 (8) | A study in Barcelona | cross-sectional | Spain | COPD patients:  patients ≥40 years of age diagnosed with COPD in medical records; smokers or ex- smokers of at least 10 pack-years; and a post-BD FEV_1_/FVC ＜ 0.7 | 3,125 | ACO definition: with COPD and a history of asthma before the age of 40 years  ACOS 1: patients that fulfilled the ACOS diagnostic criteria of the Spanish consensus. Fulfill two major, or one major and two minor criteria. 1) major criteria: very positive bronchodilator test (improvement in FEV_1_＞400 mL and＞15%); sputum eosinophilia or a previous diagnosis of asthma before the age of 40 years. 2) Minor criteria: increased total serum immunoglobulin (Ig)E; and previous history of atopy or a positive bronchodilator test (＞200 mL and＞12% in FEV_1_) on at least two occasions.  ACOS 2: the remaining patients with ACOS, diagnosed only on the basis of a history of asthma before the age of 40 years, but not fulfilling the diagnostic criteria of the Spanish consensus. | 496 | NA | | 15.9%  (ACOS 1: 5.1%,  ACOS 2: 10.8%)  (COPD+HBR1 [BDT: FEV_1_＞200 mL and＞12%]): 16.8%),  COPD+HBR2 [BDT: FEV_1_＞400 mL and＞15%]): 11.9%) |
| Romem A, 2020 (9) | A study in Jerusalem | cross-sectional | Israel | patients who were referred for PFT at the Rokach Institute | 3,669 referrals, 215 were included | ACO definition: the combination of COPD and asthma  COPD: post-BD FEV_1_/ FVC <0.70;  asthma: physician-diagnosed asthma before age 40 and/or minimum post-BD increase in FEV_1_ or FVC of 12% and 200 mL | 82 | NA | | 49.4% |
| Hayden L P, 2018 (10) | COPDGene | cohort | USA | phase 1: 10,199 current and former smokers with and without COPD  phase 2: 4,915 | phase 1: 10,199  phase 2: 4,915 | ACO: subjects with COPD who self-reported asthma diagnosed by a health professional with age of onset at ≤ 40 years or during childhood  COPD: Global Initiative for Chronic Obstructive Lung Disease (GOLD) 2007 spirometry grades 2 to 4, corresponding to a post-BD FEV_1_/ FVC ratio < 0.7 with FEV_1_ < 80% predicted | phase 1: 569  phase 2: 242 | NA | | phase 1: 15.5%  phase 2: 15.1% |
| Wurst K E, 2016 (11) | ECLIPSE study | longitude | multi-country | COPD patients  (40–75 years, with baseline post-BD FEV_1_ of 80% of the predicted value, baseline post-BD FEV_1_/FVC of ＜ 0.7 and a smoking history of o10 pack-years) | 1,976 | ACO definition: COPD answering “yes” to “Have you ever had asthma?” | 493 | NA | | 25%  (COPD+HBR[ΔFEV_1_ ⩾12% and ⩾200 mL]: 5.6%) |
| **First author, publication year** | **Study Name** | **Type of Study** | **Country** | **Population** | **Sample Size** | **ACO Definition** | **ACO Subject** | **ACO Prevalence** | | |
|  |  |  |  |  |  |  |  | **in general population** | **in COPD** | |
| Inoue H, 2017 (12) | NA | Multicenter, cross-sectional, observational study enrolled outpatients who were receiving medical treatment for COPD. | Japan | COPD patients treated by specialists (FEV_1_/ FVC＜0.7, age ≥ 40 years at the time of COPD diagnosis, ever smoker with a history of ≥ 10 pack-years) | 1,008 | Using a stepwise approach as stated in the GINA/GOLD report. 1. patients who had both three or more features favoring asthma and three or more features favoring COPD, were selected as candidates for ACOS; 2. patients who also had variable airflow limitation were identified as having ACOS (variable airflow limitation was determined based on the results of airway reversible tests in medical records); 3. In addition, a long-term airway clinical variability as a criterion for ACOS (patients who had a ≥12% and ≥ 200 mL post-BD FEV_1_ increase from baseline in the airway reversibility test and/or a difference of ≥12% and ≥200 mL between the highest and lowest FEV_1_ values in the past 3 years were identified as having ACOS with the ≥200 mL criterion; alternatively, patients who had a ≥12% and ≥400 mL post-BD FEV_1_ increase and/or a difference of ≥12% and ≥400 mL between the highest and lowest FEV_1_ values in the past 3 years were identified as having ACOS with the ≥400 mL criterion) | 93 ACOS defined by syndromic features and FEV_1_ variability ≥12% and ≥200 mL;  42 ACOS defined by syndromic features and FEV_1_ variability ≥12% and ≥400 mL | NA | ACOS were found to be 9.2% and 4.2% (depending on the different FEV_1_ variability cutoff) | |
| Izbicki G, 2019 (13) | RADICALS trial | An intervention study of an interdisciplinary community-based model of care. | Australia | COPD patients aged 40 years or older, current or ex-smokers with a history of at least 10 pack-years | From this total of 272 participants with COPD, 60 (22%) were identified to have spirometric features consistent with ACO. | ACO: COPD patients with acute bronchodilator reversibility defined as ≥ 12% and ≥ 200 mL post-BD FEV_1_ increase from baseline | 60 | NA | 22% | |
| Barrecheguren M, 2020 (14) | This study was embedded in the Canadian Cohort  Obstructive Lung Disease (CanCOLD), a prospective,  multicentre study that recruited patients with COPD  based on spirometric GOLD 1–4, at-risk individuals  (smoker with normal post-bronchodilator spirometry)  and healthy controls.  This study was embedded in the Canadian Cohort  Obstructive Lung Disease (CanCOLD), a prospective,  multicentre study that recruited patients with COPD  based on spirometric GOLD 1–4, at-risk individuals  (smoker with normal post-bronchodilator spirometry)  and healthy controls.  The CanCOLD study | A aprospective, multicentre study that recruited patients with COPD based on spirometric GOLD 1–4, at‐risk individuals (smoker with normal post‐bronchodilator spirometry) and healthy controls. | Canada | From a total of 1561 CanCOLD participant (including COPD of 719, 466 of at risk and 336 healthy controls), there were 719 individuals with COPD at baseline, of whom 522 were included in this analysis | 522 COPD patients | COPD: post‐BD FEV_1_/FVC < 0.70 within the CanCOLD population;  ACO: 1. reversibility: post‐BD FEV_1_ increased >12% and >200 mL, 2. large reversibility: post‐BD FEV_1_ increased >15% and >400 mL, 3. physician diagnosis of asthma as reported in a self‐reported questionnaire); 4. reversibility and atopy; 5. atopy and a physician diagnosis of asthma; 6. reversibility pre‐post-BD, atopy and physician diagnosis of asthma. | 264 | NA | Definition 1: 20.1%; definition 2: 5.9%; definition 3: 23.8%; definition 4: 5.2%; definition 5: 15.3%; definition 6: 3.8%; | |
| Toledo-Pons N, 2019 (15) | MAJORICA cohort | This study used a retrospective design with prospective follow-up from a health-related population database. | Spain | Individuals registered for primary care in the Balearic Islands, Spain, during 2012 were included in the cohort that contains follow-up data until 2015. | 603 patients who fulfilled all criteria, including 1. ≥ 40 years of age; 2. smoking exposure > 10 pack-years; 3. post-BD FEV_1_/FVC < 0.7; 4. at least one eosinophil count in 2014; and 5. follow-up until 2015 | ACO: COPD patients with a concomitant diagnosis of asthma; or COPD with HBR (defined as post‐BD FEV_1_ increased >400 mL and 15%; or COPD patients with blood eosinophil count greater than 300 cells/μL | 165 | NA | Total: 27.4%  Smoking asthmatics: 13.8%  COPD-HBR: 1.49%  COPD-EOS: 12.1% | |

**REFERENCES**

1. de Marco R, Pesce G, Marcon A, Accordini S, Antonicelli L, Bugiani M, et al. The coexistence of asthma and chronic obstructive pulmonary disease (COPD): prevalence and risk factors in young, middle-aged and elderly people from the general population. *PLoS One.* (2013) 8: e62985. doi: 10.1371/journal.pone.0062985

2. Guerriero M, Caminati M, Viegi G, Senna G, Pomari C. Prevalence and features of asthma-chronic obstructive pulmonary disease overlap in Northern Italy general population. *J Asthma.* (2019) 56: 27-33. doi: 10.1080/02770903.2018.1424190

3. Morgan BW, Grigsby MR, Siddharthan T, Chowdhury M, Rubinstein A, Gutierrez L, et al. Epidemiology and risk factors of asthma-chronic obstructive pulmonary disease overlap in low- and middle-income countries. *J Allergy Clin Immunol.* (2019) 143: 1598-606. doi: 10.1016/j.jaci.2018.06.052

4. Miravitlles M, Soriano JB, Ancochea J, Muñoz L, Duran-Tauleria E, Sánchez G, et al. Characterisation of the overlap COPD-asthma phenotype. Focus on physical activity and health status. *Respir Med.* 2013; 107: 1053-60. doi: 10.1016/j.rmed.2013.03.007

5. Kumbhare S, Pleasants R, Ohar JA, Strange C. Characteristics and prevalence of asthma/chronic obstructive pulmonary disease overlap in the United States. *Ann Am Thorac Soc.* (2016) 13: 803-10. doi: 10.1513/AnnalsATS.201508-554OC

6. Menezes AMB, Montes de Oca M, Pérez-Padilla R, Nadeau G, Wehrmeister FC, Lopez-Varela MV, et al. Increased risk of exacerbation and hospitalization in subjects with an overlap phenotype: COPD-asthma. *Chest.* (2014) 145: 297-304. doi: 10.1378/chest.13-0622

7. Krishnan JA, Nibber A, Chisholm A, Price D, Bateman ED, Bjermer L, et al. Prevalence and Characteristics of Asthma-Chronic Obstructive Pulmonary Disease Overlap in Routine Primary Care Practices. *Ann Am Thorac Soc.* (2019) 16: 1143-50. doi: 10.1513/AnnalsATS.201809-607OC

8. Barrecheguren M, Roman-Rodriguez M, Miravitlles M. Is a previous diagnosis of asthma a reliable criterion for asthma-COPD overlap syndrome in a patient with COPD? *Int J Chron Obstruct Pulmon Dis.* (2015) 10: 1745-52. doi: 10.2147/COPD.S87025

9. Romem A, Rokach A, Bohadana A, Babai P, Arish N, Azulai H, et al. Identification of asthma-COPD overlap, asthma, and chronic obstructive pulmonary disease phenotypes in patients with airway obstruction: influence on treatment approach. *Respiration.* (2020) 99: 35-42. doi: 10.1159/000503328

10. Hayden LP, Hardin ME, Qiu W, Lynch DA, Strand MJ, van Beek EJ, et al. Asthma is a risk factor for respiratory exacerbations without increased rate of lung function decline: five-year follow-up in adult smokers from the COPDGene study. *Chest.* (2018) 153: 368-77. doi: 10.1016/j.chest.2017.11.038

11. Wurst KE, Rheault TR, Edwards L, Tal-Singer R, Agusti A, Vestbo J. A comparison of COPD patients with and without ACOS in the ECLIPSE study. *Eur Respir J.* (2016) 47:1559-62. doi: 10.1183/13993003.02045-2015

12. Inoue H, Nagase T, Morita S, Yoshida A, Jinnai T, Ichinose M. Prevalence and characteristics of asthma-COPD overlap syndrome identified by a stepwise approach. *Int J Chron Obstruct Pulmon Dis.* (2017) 12: 1803-10. doi: 10.2147/COPD.S133859

13. Izbicki G, Teo V, Liang J, Russell GM, Holland AE, Zwar NA, et al. Clinical characteristics of patients with asthma COPD overlap (ACO) in Australian primary care. *Int J Chron Obstruct Pulmon Dis.* (2019) 14: 2745-52. doi: 10.2147/COPD.S220346

14. Barrecheguren M, Pinto L, Mostafavi-Pour-Manshadi SM, Tan WC, Li PZ, Aaron SD, et al. Identification and definition of asthma-COPD overlap: The CanCOLD study. *Respirology.* (2020) 25: 836-49. doi: 10.1111/resp.13780

15. Toledo-Pons N, van Boven JFM, Román-Rodríguez M, Pérez N, Valera Felices JL, Soriano JB, et al. ACO: Time to move from the description of different phenotypes to the treatable traits. *PLoS One.* (2019) 14: e0210915. doi: 10.1371/journal.pone.0210915
